# A Pilot Study on Early-Onset Schizophrenia Reveals the Implication of Wnt, Cadherin and Cholecystokinin Receptor Signaling in Its Pathophysiology

**DOI:** 10.3389/fgene.2021.792218

**Published:** 2021-12-17

**Authors:** Malgorzata Marta Drozd, Maria Capovilla, Carlo Previderé, Mauro Grossi, Florence Askenazy, Barbara Bardoni, Arnaud Fernandez

**Affiliations:** ^1^ Université Côte d’Azur, CNRS UMR7275, Institut de Pharmacologie Moléculaire et Cellulaire, Valbonne, France; ^2^ Laboratorio di Genetica Forense, Unità di Medicina Legale e Scienze Forensi Antonio Fornari, Dipartimento di Sanità Pubblica, Medicina Sperimentale e Forense, Università di Pavia, Pavia, Italy; ^3^ Département de Psychiatrie de l’Enfant et de l’Adolescent, Hôpitaux Pédiatriques de Nice, CHU-Lenval, Nice, France; ^4^ CoBTek, EA7276, Université Côte d’Azur, Valbonne, France; ^5^ Université Côte d’Azur, Inserm, CNRS UMR7275, Institut de Pharmacologie Moléculaire et Cellulaire, Valbonne, France

**Keywords:** Early-Onset Schizophrenia, Autism Spectrum Disorder, Intellectual Disability, WNT, Cadherin, Cholecystokinin receptor, Whole Exome Sequencing

## Abstract

Early-Onset Schizophrenia (EOS) is a very rare mental disorder that is a form of schizophrenia occurring before the age of 18. EOS is a brain disease marked by an early onset of positive and negative symptoms of psychosis that impact development and cognitive functioning. Clinical manifestations commonly include premorbid features of Autism Spectrum Disorder (ASD), attention deficits, Intellectual Disability (ID), neurodevelopmental delay, and behavioral disturbances. After the onset of psychotic symptoms, other neuropsychiatric comorbidities are also common, including obsessive-compulsive disorder, major depressive disorder, expressive and receptive language disorders, auditory processing, and executive functioning deficits. With the purpose to better gain insight into the genetic bases of this disorder, we developed a pilot project performing whole exome sequencing of nine trios affected by EOS, ASD, and mild ID. We carried out gene prioritization by combining multiple bioinformatic tools allowing us to identify the main pathways that could underpin the neurodevelopmental phenotypes of these patients. We identified the presence of variants in genes belonging to the Wnt, cadherin and cholecystokinin receptor signaling pathways.

## Introduction

Early-Onset Schizophrenia (EOS) is a very rare form of schizophrenia (SCZ) affecting children before the age of 18 (Grover and Avasthi, 2019). Very early-onset schizophrenia (also called Childhood-Onset Schizophrenia) is the rarest form of SCZ (less than 1 out of 10,000 individuals) and occurs before the age of 13 (Grover and Avasthi, 2019). According to the Diagnostic and Statistical Manual of mental disorders fifth edition (DSM-5), SCZ patients need to exhibit at least one of the following positive symptoms: delusions (firm convictions based on false information that cannot be changed when confronting reality), hallucinations (perception without a real external stimulus) or disorganized speech (*e.g.*, changing from one subject to another). Negative symptoms include lack of interest in social activities and interactions, reduction in showing feelings, low self-motivation, poverty of the speech content, and anhedonia (M-5 2020 Manuel diagnos, 2020). The diagnosis of EOS is based on the same criteria as in patients diagnosed with Adult Onset Schizophrenia (AOS), with a good long-term diagnostic stability (Maziade et al., 1996). Before the introduction of DSM-III, the diagnostic criteria for EOS were not well established (Forsyth and Asarnow, 2020). In addition, the occurrence of EOS is usually preceded by a period of normal development. Sometimes, atypical interests and beliefs can be confused with social deficits typical of Autism Spectrum Disorder (ASD) patients (Fernandez et al., 2021a). Indeed, around 27% of EOS patients, before manifestation of the first psychotic symptoms, meet criteria for ASD (Driver et al., 2020). Hallucinations and delusions are traits that can help distinguish EOS patients from ASD (M-5 2020 Manuel diagnos, 2020). Similar to other neurodevelopmental disorders, EOS is characterized by genetic heterogeneity (Fernandez et al., 2019). Genetic studies of EOS patients suggest that rare Copy Number Variations (CNVs) are more common in EOS than in AOS. Some of these rare CNVs are also associated with ASD and ID (Addington and Rapoport, 2009; Fernandez et al., 2019; Fernandez et al., 2021b). EOS is thought to have higher familial transmission and higher prevalence of rare allelic variants than AOS and was associated with common polymorphisms in *Glutamate decarboxylase 1* (*GAD1*), *Dysbindin-1* (*DTNBP1*), *Neuregulin 1* (*NRG1*), *G72/G30*, *and Brain-derived neurotrophic factor* (*BDNF*) (Fernandez et al., 2019). Genome-Wide Association Studies (GWAS) of AOS patients involving more than 16,161 individuals did not indicate significant changes. For this reason and due to the low prevalence of EOS, GWAS on EOS patients have not yet been performed (Forsyth and Asarnow, 2020). Importantly, only in one third of genetic studies related to EOS, the full phenotype of patients is described (Fernandez et al., 2019).

Due to the importance to define the genetic bases of EOS, we performed a pilot study carrying out Whole Exome Sequencing (WES) on nine EOS patients and their parents. The main objective is to identify in these patients disease-causing mutations primarily in genes involved in neurodevelopmental pathways. We carried out gene prioritization by the following multiple bioinformatic tools: Residual Variation Intolerance Score (RVIS) (Petrovski et al., 2015), Gene Demage Index (GDI) (Itan et al., 2015), STRING (Franceschini et al., 2013), IntegraGen tools, VarElect, DatabasE of genomiC variation (Stelzer et al., 2016) and Phenotype in Humans using Ensembl Resources (DECIPHER), and Protein ANalysis THrough Evolutionary Relationships (PANTHER) (Mi et al., 2020). This allowed us to identify pathways that could underpin the neurodevelopmental phenotypes of these patients and that could be useful to understand the pathophysiology of these disorders paving the way for future therapeutic interventions.

## Materials and Methods

### Trio Recruitment and Clinical Evaluations

Patients with EOS were initially recruited through the 2011–2013 Interregional Hospital Clinical Research Program (NCT01512641). The main goal of this program was to estimate the prevalence of EOS in a population of children in child psychiatric care or medico-educational structures (Dor-Nedonsel et al., 2020). In addition, an enrolment in the GenAuDiss protocol (NCT02565524) study was offered to in- and out-patients, to Child and Adolescent Psychiatry (CAP) centers, and to their first-degree relatives (Fernandez et al., 2018). Participants were included either directly at the study sites or after referral by child and adolescent psychiatrists of the Provence-Alpes-Côte d'Azur (PACA) region of south of France. At inclusion (V1), clinical assessments (both psychiatric and neurocognitive) were performed in patients as well as first-degree relatives (parents and siblings). The following quantitative and qualitative measures were assessed during the study: 1) Clinical parameters from medical history (including pregnancy and birth) and biographic parameters, types and dates of significant life events including trauma and environmental exposures (*e. g.*, drugs and substances); 2) Clinical parameters from physical examination (*e. g.*, body weight, BMI and arterial pressure); 3) Semistructured interviews to assess the main diagnosis at the time of inclusion (K-SADS-PL, ADI-R, and MINI); 4) Clinical heteroassessments with specific neurocognitive rating scales (WISC-V, TMTA, TMTB, and Verbal fluency) and specific psychiatric rating scales (PANSS and SANS); self-report questionnaires (Cloninger TCI 226 to assess personality trouble and Baron Cohen AQ to assess autistic traits). In Table 1, all the main phenotypes are recapitulated.

**TABLE 1:** Prenatal, perinatal, and neurodevelopemental phenotypes.

	Family 1	Family 2	Family 3	Family 4	Family 5	Family 6	Family 7	Family 8	Family 9
Pregnancy complications									
Maternal disorders	no	no	no	no	no	no	no	*In vitro* fertilization and	no
Drugs	no	no	no	no	no	no	no	no	no
Maternal smoking during pregnancy	no	yes	yes	no	yes	no	no	no	no
Birth characteristics									
Maternal age at birth of child (years)	21	32	32	33	26	29	34	33	34
paternal age at birth of child (years)	24	35	40	32	24	51	32	34	40
Birth weight (grams)	3,000	3,020	1,570	3,500	2,950	normal	3,180	3,280	normal
Multiple birth	no	no	no	no	no	no	no	twin	no
Method of delivery	Vaginal birth	Vaginal birth	Vaginal birth	Vaginal birth	Vaginal birth assisted by vacuum	Vaginal birth	Vaginal birth	Vaginal birth	Vaginal birth
Apgar score	10 10 10	10 10 10	10 10 10	10 10 10	09 10 10	10 10 10	10 10 10	08 10 10	10 10 10
Gestational age (weeks)	33	39	35	41	41	normal	40	39	normal
Perinatal conditions									
Infections	no	no	no	no	no	no	Neonatal bronchiolitis	no	no
Digestive, endocrine or metabolic disorders	no	no	no	no	Neonatal jaundice	no	no	no	no
Other perinatal conditions	no	no	no	no	Matermal depression	no	no	Single nuchal cord	no
Congenital malformation									
	no	no	no	no	no	no	no	no	no
Neurodevelopemental impairements									
Intellectual ability (IQ)	96	73	53	105	47	40	69	63	71
Communication	no	no	yes	no	yes	yes	yes	yes	yes
Motor	no	no	no	no	no	no	no	yes	yes
Learning	no	yes	yes	yes	yes	yes	yes	yes	yes
ADHD	no	yes	no	no	yes	yes	yes	yes	yes
ASD	yes	no	yes	no	yes	no	yes	yes	yes
Consanguinity									
	no	no	yes	no	no	no	no	no	no

### Sample Collection

Blood samples of trios (patients and their parents) were collected by the staff of the University Children’s Hospital of Nice (France). Samples were anonymized and DNA from blood was extracted using the GenElute Mammalian Genomic DNA Miniprep Kit (SIGMA Cat. No. G1N70). First, we performed current testing (including DNA testing for Fragile-X Syndrome, high-resolution karyotype and CGH-array). Then, in case of negative results from this step, we performed WES on trios. Isolated DNA samples were sent for WES analysis to IntegraGen SA (Evry, France).

### Targeted Exome Sequencing

Library preparation, exome capture, sequencing and data analysis were done by IntegraGen SA (Evry, France). Genomic DNA was captured using Agilent in-solution enrichment methodology (SureSelect SureSelect XT Clinical Reasearch Exome, Agilent) with their biotinylated oligonucleotides probes library (SureSelect XT Clinical Reasearch Exome—54 Mb, Agilent), followed by paired-end 75 bases massively parallel sequencing on an Illumina HiSeq4000 instrument.

Sequence capture, enrichment and elution were performed according to the manufacturer’s instruction (SureSelect, Agilent) without modification except for library preparation performed with the NEBNext^®^ Ultra kit (New England Biolabs^®^). For library preparation 600 ng of each genomic DNA were fragmented by sonication and purified to yield fragments of 150–200 bp. Paired-end adaptor oligonucleotides from the NEB kit were ligated on repaired, tailed fragments then purified and enriched by 8 PCR cycles. 1,200 ng of these purified libraries were then hybridized to the SureSelect oligo probe capture library for 72 h. After hybridization, washing and elution, the eluted fraction was PCR-amplified with 9 cycles, purified and quantified by qPCR to obtain sufficient DNA template for downstream applications. Each eluted-enriched DNA sample was then sequenced on an Illumina HiSeq4000 as paired-end 75 base reads. Image analysis and base calling were performed using Illumina Real Time Analysis (2.7.3) with default parameters.

Base calling was performed using the Real-Time Analysis software sequence pipeline (2.7.3) with default parameters. Sequence reads were mapped to the human genome build (hg19/GRCh37) using Elandv2e (Illumina, CASAVA1.8.2) allowing multiseed and gapped alignments. Duplicated reads (*e.g.*, paired-end reads in which the insert DNA molecule showed identical start and end locations in the human genome) were removed.

CASAVA1.8.2 was used to call single-nucleotide variants (SNVs) and short insertions/deletions (max. size = 300 nt), taking into account all reads per position. SNVs and indels with Q (SNPs) < 10 and Q (Indel) < 20 or regions with low mappability (QVCutoff <90) were filtered out. The frequency with which single base differences are expected between two unrelated haplotypes (Theta parameter) is 0.01. This frequency was set to 0.001 for indels.

Variant annotation takes into account data available in dbSNP (dbSNP144), the 1,000 Genomes Project (phase1_release_v3.20101123), the Exome Variant Server (ESP6500SI-V2-SSA137), and the Exome Aggregation Consortium (ExAC r3.0) and from an in-house database (201 exomes whole exomes for SNVs and 130 exomes whole exomes for indels). Functional consequences of variants on genes, transcripts and protein sequence, as well as regulatory regions, are predicted by Variant Effect Predictor (VEP release 83) (stop, splicing, missense, synonymous … ), as well as by location of the variants (*e.g.,* upstream of a transcript, in coding sequence, in non-coding RNA, in regulatory regions). Regarding missense changes, two bioinformatic predictions for pathogenicity were used: SIFT (sift5.2.2) and PolyPhen (2.2.2). Other informations like quality score, homozygote/heterozygote status, count of variant allele reads, the presence of the variant in the COSMIC database (version71) were reported.

To investigate genomic copy number aberrations (CNA) (*e.g.*, copy number gains and copy number losses), we used the Bioconductor DNACopy package (DNAcopy 1.32.0) by comparing the normal DNA exome data to a reference sample pool. It implements the Cystathionin Beta-Synthase algorithm to segment DNA copy number data. All changes were annotated with the catalog of the Database of Genomic Variants (DGV) to provide a comprehensive summary of structural variation in the human genome.

### Bioinformatic Analysis

Gene prioritization was performed by combining the multiple bioinformatic tools we describe here. RVIS is based on the assumption that genes with common genetic functional variations are more tolerant and the variations occurring in these genes are common (higher RVIS score) and are unlikely to be disease-causing. On the other hand, genes with lower genetic variation (lower score) are considered to be intolerant and have high chances to be disease-causing. The RVIS percentile refers to the percentage of intolerant genes among which the gene of interest is found (Petrovski et al., 2015). GDI predicts if a given gene is prone to harbor disease-causing variants. The index evolved from the observation that more than a half of rare variants found in protein coding sequences are located in 2% of the genes (Itan et al., 2015). STRING is a protein-protein interaction database that selects data based on experiments (co-crystallization, co-purification and genetic interaction), databases (pathways and protein complexes), textmining (names of proteins that are often mentioned together), co-occurrence of gene patterns across genomes, and coexpression (Franceschini et al., 2013). VarElect is a tool that enables gene prioritization according to the disease and/or phenotype association. It also includes access to tools such as RVIS and GDI (Stelzer et al., 2016). DECIPHER is a database that combines multiple tools to facilitate evaluation of the impact of a given variant (Chatzimichali et al., 2015). The PANTHER classification system facilitates protein and gene categorization according to family and subfamily classes, molecular function, biological process, and pathways (Mi et al., 2020). MutationTaster enables to estimate the pathogenic potential of DNA sequence variants (Schwarz et al., 2014).

### Statistical Analyses

The two tails Mann-Whitney Test was performed for all the analyses. Data are presented in the form of violin plots. Statistical analyses were performed using the Prism Software 7 and 8 versions (GraphPad Software, Inc.).

### Ethic Statement

The study protocol was approved by the Local Ethic Committee “Sud Méditerranée V” (number 14.002) and authorized by the French National Agency for Medicines and Health Products Safety (ANSM 2013-A01699-36). All patients signed a document authorizing the analyses described in this study. Collection of patients was authorized for a precise period (April 2014 - November 2021) and it was registered on ClinicalTrials.gov (NCT02565524).

## Results

### De novo Variant Analysis

**FIGURE 1:**
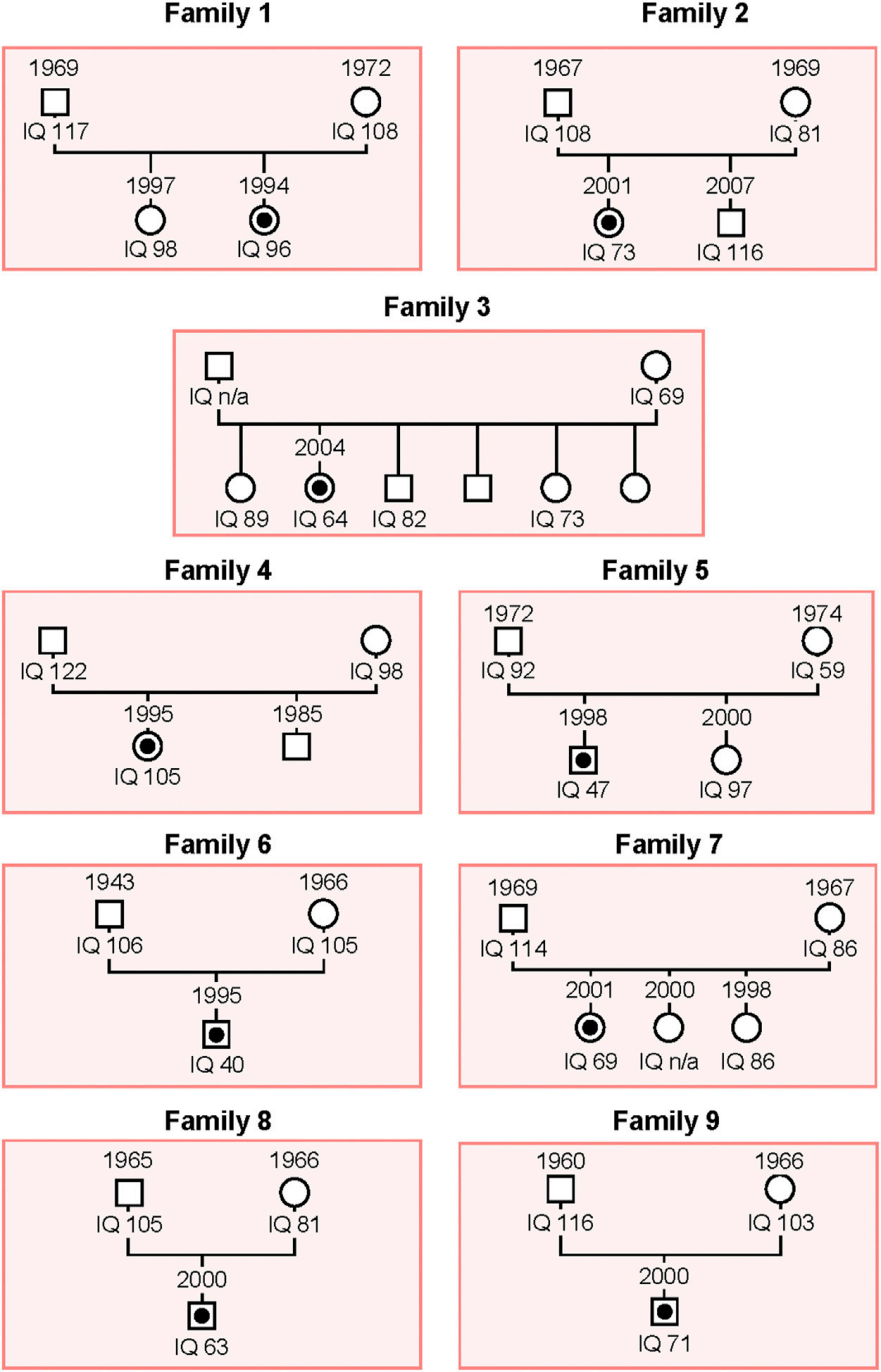
Schema of the genealogical trees of Families 1–9. The proband of each family is indicated with a black dot. Squares: men; circles: women.

As described in the Material and Methods section, we carried out WES on patients belonging to nine unrelated families whose genealogic trees are described in Figure 1.

**FIGURE 2:**
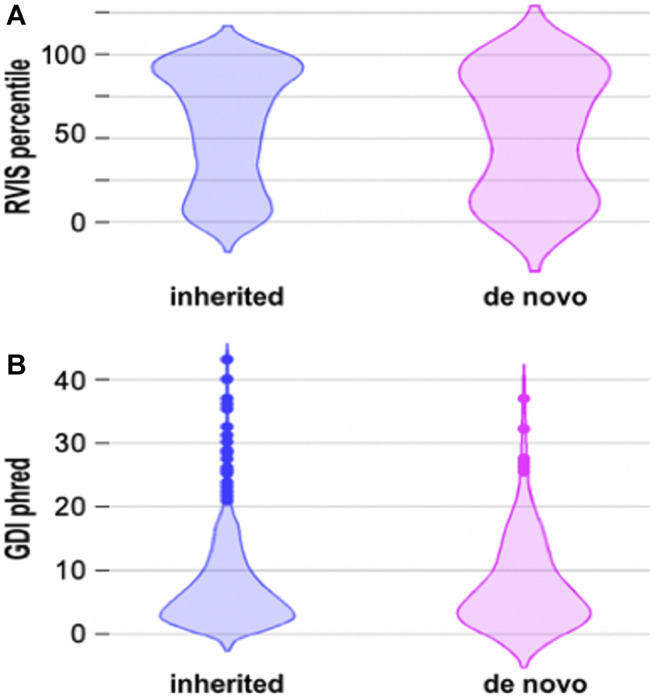
Voilin plots for RVIS and GDI. **(A)**. Violin plots showing the RVIS percentile. Lower percentile indicates more “intolerant” variants. Statistical analysis with the two-tails Mann Whitney test did not reveal statistical differences between inherited and *de novo* mutations. **(B)**. Voilin plot showing the GDI phred. Lower GDI values indicate genes more prone to har-bour disease-causing mutations. Statistical analysis with the two-tails Mann Whitney test did not reveal statistical differences between inherited and *de novo* mutations.

Previous data suggest that there is a difference between the RVIS and GDI scores of inherited and *de novo* mutations. *De novo* mutations are thought to occur more frequently in genes that are more prone to harbor disease-causing variants and have less common variation (Ambalavanan et al., 2016). We compared the RVIS and GDI scores of inherited and *de novo* mutations, but statistical analysis with the two tails Mann-Whitney Test did not reveal statistical differences between these two groups (Figure 2). Even when we considered only missense *de novo* mutations (Supplementary Table S1), we did not obtain the same pattern as in previous studies (Figure 2). Actually, more missense variants occurred in genes that were predicted to have more common variation (Supplementary Table S1), which is opposite to what was found by other groups (Ambalavanan et al., 2016).

In order to find a link between trios, we created a Venn Diagram with the *de novo* variants identified in families 1–9 (Supplementary Figure S1). We focused on exon and splicing intron sequences, taking into consideration missense, nonsense plus splice exon, frameshift, in frame deletion/insertion, and splice acceptor variants. Due to splice variant identification, some changes were localized in the intronic part of the genes. We focused on *de novo* variants in genes that were common for at least three families from our dataset (Supplementary Table S2).

In families 1-5 and 7, we identified many *de novo* changes in *Zinc Finger Protein 717* (*ZNF717*) that is a transcriptional regulator coding the KRuppel-Associated Box (KRAB) zinc-finger protein. According to the DECIPHER database, *ZNF717* was found within the CNV region in five patients suffering from ID and in two patients with ASD. All reference SNPs (rs2918517, rs3009004, rs78135954, rs80214016, rs74357986, rs77110669, rs79138891, rs73843014, rs75737034, rs150497643, rs149076283 rs77378861, and rs776210532) do not have ClinVar reference. Most altered alleles have low frequency or data are not available. GDI predicts that changes within the *ZNF717* gene have moderate chances to be disease-causing. In 2018, a *de novo* variant in *ZNF717* was reported as a mutation potentially associated with growth retardation and ID (Diao et al., 2018).

Variants in Mucin 6 (MUC6) were identified in families 1–5. MUC6 is actually one of the most frequently mutated genes in the general population and are susceptible to false positive results in next generation sequencing http://massgenomics.org/2013/06/ngs-false-positives.html (Itan et al., 2015). However, evidence of late-onset Alzheimer disease (LOAD)-associated genetic polymorphism within an exon of Mucin 6 (MUC6) and immediately downstream from the Adaptor Related Protein Complex 2 Subunit Alpha 2 (AP2A2) have been reported (Katsumata et al., 2020).

Variants of the *FSHD Region Gene 1* (*FRG1*) gene were found in Families 1–3. *FRG1* (located in 4q35) codes a splicing factor and was implicated in FascioScapuloHumeral muscular Dystrophy (FSHD) as a modifier gene (DeSimone et al., 2017) (Jagannathan et al., 2019). 4p/4q CNVs of variable sizes have been associated with several different psychiatric findings and developmental disability. It was shown that the prevalence of independent deletions at 4p/4q involves PIGG, TRIML2, and FRG1. This suggests a possible implication of FRG1 in neurodevelopmental disorders (Novo-Filho et al., 2016). This gene is also susceptible of false positive results (http://massgenomics.org/2013/06/ngs-false-positives.html).

Another variant shared by families 1–3 is *Poly(A) Binding Protein Cytoplasmic 1* (*PABPC1*). This gene was shown to be upregulated in male patients affected by SCZ (Qin et al., 2016). According to GDI prediction, *PABPC1* has moderate chances to harbor disease-causing mutations. On the other hand, RVIS predicts that this gene has less common genetic variation.

Variants in *Aggrecan* (*ACAN*) were found in families 2, 3, and 4. The variant found in family 4 (rs12899191) turned out to have a high frequency in the general population [G = 0.27107 (3,538/13,052, GnomAD)] and is not reported in the ClinVar database. DECIPHER analysis shows that this gene is extremely intolerant to loss-of-function variation (Burd and Kerbeshian, 1987) (Gochman et al., 2011). *ACAN* was shown to be downregulated in the brain cortex region of SCZ individuals (Pietersen et al., 2014). Both RVIS and GDI predict this gene as highly mutated which means that there is little probability to be a disease-causing gene. A same kind of variant in *RNA-Binding Motif Protein, X-Linked-Like-3* (*RBMXL3*), an RNA binding protein with elevated recurrent mutation rate in neuroblastoma (Lee et al., 2020), was found in families 1, 3, and 4. The DECIPHER database provides reports of two patients with abnormalities in the nervous system who carry variants in the *RBMXL3* gene. MutationTaster shows that deletions in this gene might be disease-causing. GDI places this gene among ones that have moderate chances to harbor disease-causing mutations. The RVIS database does not contain information about this gene.  In order to group the genes harboring *de novo* variants according to their association with certain diseases or phenotypes, we carried out analysis with the VarElect tool. As a query, we used the following phenotypes: SCZ, ASD, ID, and neurodevelopmental disorders. We concentrated on the first 10 best scored genes (Table 2).

**TABLE 2:** The first 10 best scored genes according to VarElect predictor. SCZ, Schizophrenia; ASD, Autism Spectrum Disorder; ID, Intellectual disability.

Symbol	Gene name	Matched phenotypes	Matched phenotypes	LOG10(P)	Score	Average	RVIS	GDI
						Disease		
						Causing		
						Likelihood		
KCNB1	PotassiumVoltage-Gated Channel Subfamily B Member 1	SCZ, ASD, ID	5	2.27	69.28	62.07%	−0.05 (50.34%)	Medium
HLA-DRB1	Major Histocompatibility Complex, Class II, DR Beta 1	SCZ, ASD, ID	5	2.25	68.54	0.99%	2.97 (99.19%)	High
BPTF	Bromodomain PHD Finger Transcription Factor	ASD, ID	4	2.27	62.32	67.63%	−2.55 (0.86%)	Medium
HERC2	HECT And RLD Domain Containing E3 Ubiquitin Protein Ligase 2	SCZ, ASD, ID	5	2.1	59.22	56.87%	−5.99 (0.05%)	Medium
CBS	Cystathionine BetaSynthase	SCZ, ASD, ID	5	1.52	34.34	54.73%	−0.8 (12.53%)	Medium
NOTCH4	Notch Receptor 4	SCZ, ASD, ID	5	1.43	30.96	20.47%	0.15 (64.33%)	High
FOLH1	Folate Hydrolase 1	SCZ, ASD, ID	4	1.19	19.64	30.23%	1.33 (94.21%)	Medium
FLG	Filaggrin	ASD, ID	4	1.11	16.91	0.09%	24.3 (99.99%)	High
ASCL1	Achaete-Scute Family BHLH Transcription	SCZ, ASD, ID	4	1.09	16.51	69.21%	NA (NA)	Medium
	Factor 1							
ZNF148	Zinc Finger Protein 148	ID	3	1.16	16.04	92.24%	−0.54 (20.54%)	Medium

A missense mutation in the *Potassium Voltage-Gated Channel Subfamily B Member 1* (*KCNB1*) gene was found in a patient of family 6. This mutation is predicted by the IntegraGen tool to have a moderate impact. SIFT and PolyPhen predict this change as deleterious (score 0) and probably damaging (score 0.999), respectively. MutationTaster indicates this change as disease-causing. *KCNB1* is mainly linked to Early Infantile Epileptic Encephalopathy (de Kovel et al., 2017). The proband was, apart from EOS, diagnosed with idiopathic epilepsy, thus, this mutation identified in KCNB1 may be associated with the epileptic phenotype of the patient.

The second gene predicted by VarElect is *Major Histocompatibility Complex, Class II, DR Beta 1* (*HLA-DRB1*). A missense variant (rs201929247) in this gene was identified in family 4. MutationTaster predicts this variant to be of polymorphic nature. Moreover, this variant turns out to be very frequent [A = 0.147,043 (14,841/100,930, ExAC)] and is not reported in ClinVar. According to GDI and RVIS predictions, variations in this gene are very common. For these reasons, its impact on the phenotype is very dubious.

A variant for the third predicted gene *Bromodomain PHD Finger Transcription Factor* (*BPTF*) was identified in family 8. *BPTF* is associated with a neurodevelopmental disorder characterized by ID, dysmorphic facies and distal limb anomalies (Midro et al., 2019). The IntegraGen tool predicts the impact of this change (in-frame insertion) as moderate. GDI analysis suggests that *BPTF* is among moderately-damaging genes. RVIS predicts this gene as a gene of less common variation.

A missense variant identified in *HECT* And *RLD Domain Containing E3 Ubiquitin Protein ligase 2* (*HERC2*) was found in family 1. Mutations in HERC2 were associated with developmental delay with Angelman-like features (including severe ID) (Harlalka et al., 2013). This variant (rs146883683) is known to have high frequency [G = 0.11394 (9,547/83,792, ExAC)] and is not reported in the ClinVar database. Moreover, MutationTaster predicts this variant as polymorphism.

A missense variant in *Cystathionin Beta-Synthase* (*CBS*) was identified in family 3. According to ClinVar, the variant rs5742905 is associated with homocystinuria. Interestingly, this disorder is frequently accompanied by ID and *CBS* is one of the genes associated with Down Syndrome (Pogribna et al., 2001), (Marechal et al., 2019). MutationTaster predicts this change as disease-causing. GDI analysis suggests that *CBS* is among moderately damaging genes and RVIS predicts this gene as a gene of less common variation.

An insertion in *Neurogenic Locus Notch Homolog Protein 4* (*NOTCH4*) was identified in family 4. MutationTaster predicts this variant as a polymorphism. Interestingly, polymorphic variants in this gene were previously associated with SCZ and schizoaffective disorder (Ujike et al., 2001), (Zhang et al., 2015). Both GDI and RVIS predictions indicate that variants in this gene are not prone to be disease-causing.

A missense variant in *Folate Hydrolase 1* (*FOLH1*) was found in family 3. This variant (rs75111588) is frequent in the general population [A = 0.187,153 (20,786/111,064, ExAC)]. Hence, it is unlikely that this variant is responsible for the phenotype of the patient.

A missense variant in *Filaggrin* (*FLG*) was identified in family 5. It turned out to be frequent in the general population [G = 0.08408 (1,087/12,928, GO-ESP; G = 0.117 (70/600, NorthernSweden)], which suggests that this variant does not impact the phenotype. FLG mutations were associated with athopic dermatitis with increased ischemic stroke risk in the general population (Varbo et al., 2017).

An in-frame deletion in *Achaete-Scute Family BHLH Transcription Factor 1* (*ASCL1*) was identified in family 6. The IntegraGen tool predicts this change to have a moderate impact. GDI places this gene among the genes with moderate chances to harbor disease-causing variants. *ASCL1* promotes neural differentiation (Castro et al., 2011) and its level is more elevated during neural differentiation in murin *Fmr1*-null embryonal stem cells compared with WT cell line (Khalfallah et al., 2017). According to the genomAD browser this variant is quite frequent (delGCA = 0.01150; 340/29,556). MutationTaster predicts this change as potential polymorphism.

In family 6, we also identified a frameshift variant in *Zinc Finger Protein 148* (*ZNF148*). IntegraGen predictions indicate high impact of this variant. Mutations in *ZNF148* were associated with Global Developmental delay, Absent or hypoplastic Corpus Callosum and dysmorphic Facies (GDACCF). Apart from this disorder, the phosphorylation level of ZNF148 was shown to be decreased in SCZ patients, but its overall level of proteins was unchanged (Jaros et al., 2012).

In general, the VarElect analysis has provided a further insight into genes that are already implicated in the pathogenesis of neurological disorders. We could find interesting variants in genes such as *ZNF148* and *KCNB1* that could impact the phenotype of the proband of family 6. We were able to exclude some variants of polymorphic nature that turned out to be common in the general population and that were not associated with any neurological disorder. We came across polymorphisms that could increase the risk of SCZ (*NOTCH4*). The VarElect analysis enables to extract variants that sometimes can be overlooked in general analyses (*e.g.,* some polymorphisms that are frequently excluded from candidate genes).

### Inherited Variant Analysis

We created a Venn Diagram for inherited variants to find genes that are common among at least three families (Supplementary Figure S2). We found shared inherited variants in the following genes: *ZNF717, FAM182B, LRP5L, HRNR, OR4C5, APOL3, BCLAF1, FLG, RP1L1, MUC6, HYDIN, PLEC, TTN, NRAP, ADAMTS8, SSPO, FAM104B, DAAM2, ZFHX3,* and *AHNAK2*. According to GDI and RVIS predictions, most of these genes are less prone to harbor disease-causing mutations than the genes cited above (*de novo* variants) and belong to genes that have more variability.

We focused on genes that did not have RVIS references. In families 1 to 7, we found 17 missense variants, two frame-shifts and one insertion in *ZNF717*. This gene was previously found during *de novo* mutation analysis. Considering the high amount of variants found and the fact that the majority of them have references in SNPdb, variants in this gene may be very common and not prone to be disease-causing.

In families 3 and 4, we found variants in *Family With Sequence Similarity 182 Member B* (*FAM182B*). In family 4, we found a variant in the 5′-UTR and a missense variant. In family 3, we found a variant in the 5′-UTR and one synonymous variant. This gene is predicted by GDI to have medium chances to harbor disease-causing mutations.

In families 1, 2, and 9, we identified variants in *Hornerin* (*HRNR*). All of them have references in SNPdb (rs79513582, rs76694305, rs61814943, and rs61814936) and have high frequencies in the general population.

We found some variants in *Olfactory Receptor Family 4 Subfamily C Member 5* (*OR4C5*): 1) the same deletion (rs66829866) in families 1 and 3 was reported in SNPdb and is known to have a high frequency; 2) an insertion in family 1 (rs114053360) that has low frequency and no report in ClinVar; 3) an insertion in family 4. *OR4C5* is a gene predicted by GDI to be highly damaging.

We identified three variants in *Plectin* (*PLEC*). A missense variant in family 2 is predicted by PolyPhen2 to be benign (score 2). In family 4, we found a synonymous variant (rs113133985) described in SNPdb with low frequency. A missense variant in family 5 also turned out to have low frequency and no records in the ClinVar database. GDI was predicted to be high, whereas RVIS was very low.

Overall, all inherited variants that we found seem to have benign consequences.

### Interaction and Pathway Analysis

We carried out interaction analysis with the STRING database (Supplementary Table S3). Due to the fact, that some mothers from our cohort have mild symptoms associated with neurodevelopmental disorders, we decided to focus on the genes inherited from the mothers of the probands. We took into consideration all inherited genes regardless of their potential impact of the carrying variants. The analysis of the mutated genes that were inherited from the mother revealed that “the network among the genes subjected to the analysis had significantly more interactions than expected” (*p* = 7.08 × 10^−6^). This means that these proteins have more interactions among themselves than what would be expected for a random set of proteins of similar size drawn from the genome. Such an enrichment indicates that the proteins are at least partially biologically connected. Molecular functional enrichment in this network includes transferase activity [False Discovery Rate (FDR) = 0.00019], catalytic activity (acting on protein, FDR = 0.0011), adenyl ribonucleotide binding (FDR = 0.0011), ATP-binding (FDR = 0.0011), and ubiquitin protein ligase activity (FDR = 0.0014). For instance, among the genes in the enrich pathways we came across POLA1, PARP1, and CDK5 (full gene list in Supplementary Table S1). Interestingly, POLA1 was recently associated with X-Linked ID associated with severe growth retardation, microcephaly, and hypogonadism (Van Esch et al., 2019). Elevated levels of PARP1 have been associated with Down Syndrome which is characterized by ID (Salemi et al., 2014) and it was involved in the context of dyslexia and neuronal migration (Tapia-Páez et al., 2008). Dysregulations of the CDK5 activator are associated with SCZ (Engmann et al., 2011). Considering PFAM protein domains, enrichment was observed in protein kinase (FDR = 0.0081), protein tyrosine kinase (FDR = 0.0089), microtubule-binding (FDR = 0.0439), and kinesin motor domains (FDR = 0.0439).

We performed pathway analysis with PANTHER for altered genes that were inherited from the mother. We looked for genes that belong to the same pathways. We assigned each gene to the corresponding pathway according to PANTHER classification. Subsequently, a Venn diagram was created in order to see if the pathways co-occur in our cohort of families (Supplementary Figure S3). Functional classification analysis showed that all nine families share variants in genes belonging to the WNT and Cadherin pathways (Supplementary Figure S3). In addition, families 1 to 8 share additionally the cholecystokinin receptor (CCKR) signaling pathway. Cholecystokinin activates the CCK1 receptor that belongs to G-protein-coupled receptors. Variants associated with these pathways by PANTHER are comprised, among others, in the following genes: CDH11, CDH3, CDH18, PCDH15, PCDHA4, FAT4, FAT2, NFATC2, WNT6, WNT11, and CTNNA3.

## Discussion

In this pilot study, we carried out a WES analysis on a group of patients and their parents with the final purpose to define the possibility that deregulated molecular pathways could affect the severe phenotype of the probands. Sanger sequencing was not performed for *de novo* variants as we can now consider that this verification method is not very useful for high‐ quality SNV identification (Arteche-López et al., 2021). Even if we can not exclude the hypothesis that minor changes in these genes could contribute to the phenotypes of patients if they are co-occurring, the analysis of common genes for all the families’ studies did not reveal any strong candidate genes. Although *de novo* variants are now considered to explain some of the complex heritability of neuropsychiatric disorders such as ASD, ID and SCZ (Ambalavanan et al., 2016), the *de novo* mutations we detected were never linked to COS or AOS in previous family or GWAS studies. Only rs5742905 (*CBS*) was previously associated with hyperhomocysteinemia (which is associated with ID) in numerous studies and possibly associated with Bipolar Disorder in only one study (Permoda-Osip et al., 2014).

We reasoned that it could be interesting to analyze the interaction among deregulated pathways since genes associated with these signaling pathways may be of importance in the context the phenotype of the patients. This analysis does not aim at finding any particular disease-causing gene, but at the identification of processes and networks that could be crucial in the development of the SCZ/ASD/ID pathophysiology. Our work defined the following pathways as deregulated by the presence of shared variants in disorder-candidate genes that were never associated before to EOS: WNT, cadherin signaling and CCK1 receptor signaling. The WNT pathway participates in many processes such as regulation of gene transcription, apoptosis, proliferation, cell migration, and cytoskeletal dynamics. It plays an important role in Central Nervous System (CNS) development, in particular, in the differentiation of neural progenitors, synaptogenesis, neuronal migration, and plasticity (Mulligan and Cheyette, 2017). Aberrations in WNT signaling are related to the pathogenesis of diverse neurodevelopmental diseases such as SCZ, ASD and Bipolar Disorder (BD) (Mulligan and Cheyette, 2017). Indeed, genes such as the *Adenomatosis Polyposis Coli (APC)*, *chromodomain helicase DNA-binding protein 8* (*CHD8*), *Disrupted in schizophrenia 1* (*DISC1*), *Phosphatase And Tensin Homolog* (*PTEN*), *WNT Family Member 1* (*WNT1*), *WNT2, WNT3, WNT7A*, *β-catenin* (*CTNNB1*), *Transcription Factor 4* (*TCF4*), and *TCF7* have been linked to ASD. Wnt/β-catenin pathway loci were identified in the genomic analysis of SCZ patients and some of them were suggested to be a risk factor for this disorder. Among them: *CHD8, CTNNB1, DISC1, TCF4*, *Dickkopf WNT Signaling Pathway Inhibitor 1* (*DKK1*), and *DKK4* (Mulligan and Cheyette, 2017). It was shown that the mRNA expression levels of the WNT signaling members *Frizzled Class Receptor 7* (*FZD7*) and *Nuclear Factor Of Activated T Cells 3* (*NFATC3*) are increased in SCZ patient (Hoseth et al., 2018a) (Hoseth et al., 2018b). Moreover, the plasma levels of Dickkopf-1 and Sclerostin are lower in SCZ patients (Hoseth et al., 2018a) (Hoseth et al., 2018b). One of the best targets of Fragile X mental retardation Protein (FMRP)—whose absence causes the Fragile X Syndrome (FXS) is the mRNA of *Apc* (Maurin et al., 2018) (Sawicka et al., 2019). It is interesting to underline that some antipsychotic drugs used to treat patients affected by various forms of neurodevelopmental disorders (*e.g.*, haloperidol, clozapine, fluoxetine, and Ritalin) are WNT pathway modulators (Bae and Hong, 2018). Furthermore, lithium is known to be used in the treatment of ASD (Mintz and Hollenberg, 2019) and BD (Berk et al., 2017) and was proposed at the preclinical level for FXS, since several phenotypes of adult *Fmr1*-KO mice are rescued by lithium treatment (Berk et al., 2017). Lithium is known to act as an activator of the WNT pathway, suggesting that down-regulation of WNT signaling contributes to the pathophysiology of FXS and other forms of ASD (Liu and Smith, 2014).

The Cadherin signaling pathway, involving many cell adhesion molecules, plays a role in CNS development and is associated with WNT. Indeed, cadherins negatively regulate WNT signaling by sequestering β-catenin and play an important role in the regulation of β-catenin-dependent transcription (Howard et al., 2011). Cadherins participate in neural circuit formation, synapse development, differentiation of grey matter, neuronal migration, and spine morphology. Cadherins display spatio-temporal specific expression in the brain (Redies et al., 2012). GWAS studies revealed many genes involved in this pathway involved in neuropsychiatric diseases such as ASD, SCZ, epilepsy, BD, and ID. Cadherins have been associated to brain disorders including ID, ASD, and SCZ (Taylor et al., 2020), (Ballaz, 2017).

The CCK1 receptor is responsible for regulation of serotonin neuron activity and maintenance of proper dopamine levels in raphe nuclei (clusters of cell groups localized in the brainstem). It also influences hormone and neurotransmitter regulation in the hypothalamus. Due to its functions, CCK was suggested to be a target in the treatment of SCZ, mood disorders and drug addiction (Ballaz, 2017). Especially, the relationship between CCK and dopaminergic circuits was proposed to play a role in the development of SCZ. The concentration of CCK was shown to be lowered in some brain regions of SCZ untreated patients, leading to the conclusion that CCK may display antipsychotic effects in sub-populations of SCZ patients. Lastly, antipsychotic drug treatment can boost CCK and CCKR levels (Wang et al., 1985).

All the pathways we identified are known to be involved in ID and ASD as well as in SCZ, reinforcing the idea that a link exists among these disorders (Curley and Lewis, 2012; Zhang et al., 2014; Kwan et al., 2016; Hoseth et al., 2018a; Hoseth et al., 2018b). To date, these pathways have not been described in the context of EOS. Nevertheless, the presence of numerous variants in genes belonging to these pathways suggests a common genetic background among EOS and other neurodevelopmental disorders such as SCZ, ASD, and ID. The link between these disorders is not very surprising since epidemiological studies show co-occurrence of several neurological and psychiatric features in most neurodevelopmental disorders. This phenotypic overlap should be also mirrored at the genetic and molecular levels as a continuum of dysfunction(s) during brain development in these disorders. If EOS and SCZ are caused by the same altered pathways we can speculate that the early onset may be related to environmental factors or it is caused by the presence of additional mutations in one or more genes that “per se” is/are not causing a disease but worsen a pathology caused by other factors. In this work, we have listed all the variants identified in the patients even if some of them should be considered with caution due to their high frequency of mutation.

To allow comparison between the genetic and molecular bases of the various forms of SCZ, the accuracy of diagnoses seems to be a key point to be able to define the precise and complete phenotype of patients and their families underlying the exact age of onset of the disorder.

After this pilot study, we can conclude that for future studies of these complex diseases it will be crucial to move to larger cohorts of patients and to focus on familial analyses enlarging both phenotypic and genetic studies to other members of the family, even when they display very mild phenotypes.

## Data Availability

The datasets presented in this study have been submitted to dbGaP and can be requested to fernandez.a@pediatrie-chulenval-nice.fr.

## Supplementary Material

Supplementary Figure S1 Venn diagram showing the genetic overlap between the de novo mutations of the nine families.

Supplementary Figure S2 Venn diagram showing the genetic overlap among inherited mutations of the nine families.

Supplementary Figure S3 Venn diagram showing the genetic overlap among altered pathways of the nine families.
