# Supplementary figures and images for: A Pilot Study on Early-Onset Schizophrenia Reveals the Implication of Wnt, Cadherin and Cholecystokinin Receptor Signaling in Its Pathophysiology
Source: Front Genet. 2021 Dec 17;12:792218. doi: 10.3389/fgene.2021.792218 (PMC8719199; doi:10.3389/fgene.2021.792218)

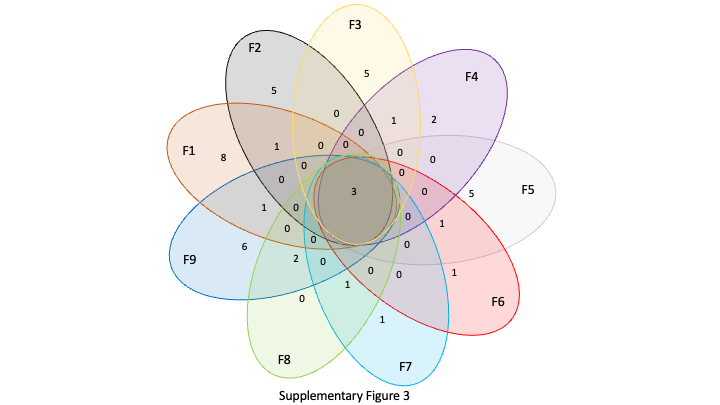

Supplement: Supplementary file 1 [file Image3.TIFF]

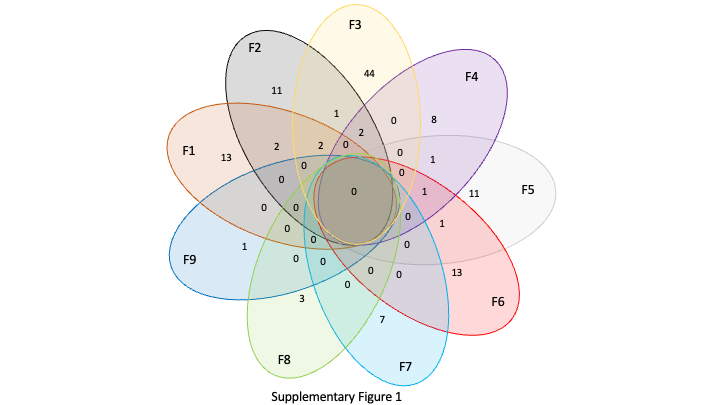

Supplement: Supplementary file 2 [file Image1.TIFF]

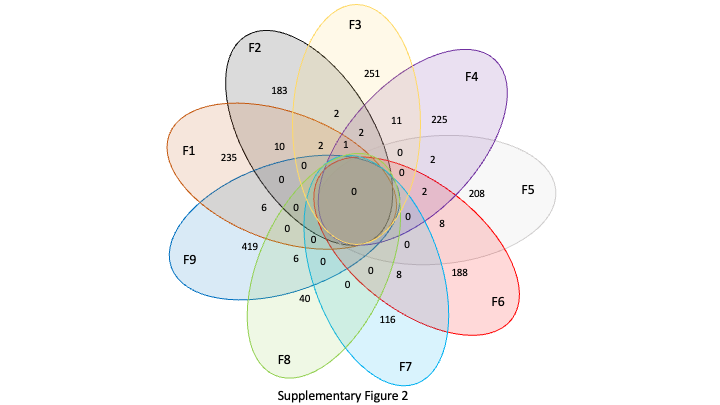

Supplement: Supplementary file 6 [file Image2.TIFF]
