# Supplementary material for: A Pilot Study on Early-Onset Schizophrenia Reveals the Implication of Wnt, Cadherin and Cholecystokinin Receptor Signaling in Its Pathophysiology
Source: Front Genet. 2021 Dec 17;12:792218. doi: 10.3389/fgene.2021.792218 (PMC8719199; doi:10.3389/fgene.2021.792218)
Supplement: Supplementary file 3 [file Table1.DOCX]

| Chr | Position | Gene | Ref seq | Protein variation | Variation type | rs | GnomAD | 1000 genomes | CADD PHRED | REVEL |  |  |  |  |  |  |  |  |
| --- | --- | --- | --- | --- | --- | --- | --- | --- | --- | --- | --- | --- | --- | --- | --- | --- | --- | --- |
| chr15 | >89399701" | ACAN | NM_001135 | D1295E | missense |  | - | - | 0.027 | 0.291 |  |  |  |  |  |  |  |  |
| chr15 | >89399758" | ACAN | NM_013227 | D1314E | missense |  | - | - | 0.008 | 0.233 |  |  |  |  |  |  |  |  |
| chr15 | >89400023" | ACAN | NM_013227 | T1403A | missense | rs12899191 | 0.0828 | - | 0.006 | 0.197 |  |  |  |  |  |  |  |  |
| chr19 | >17397483" | ANKLE1 | NM_001278444.1 | V639L | missense | rs367668712  &rs60338123 | 0.1654 | - | 7.939 | - |  |  |  |  |  |  |  |  |
| chr2 | >96617102" | ANKRD36C | ENST00000528268 | E420K | missense |  | - | - | - | - |  |  |  |  |  |  |  |  |
| chr2 | >96604606" | ANKRD36C | ENST00000456556 | G535V | missense | rs80350011 | 0.1627 | - | 9.373 | 0.031 |  |  |  |  |  |  |  |  |
| chr9 | >33385656" | AQP7 | NM_001170 | Q245L | missense |  | - | - | - | - |  |  |  |  |  |  |  |  |
| chr21 | >11049596" | BAGE2 | NM_182482 | C102Y | missense | rs28571918 | 0.00872 | - | 14.77 | - |  |  |  |  |  |  |  |  |
| chr16 | >90095620" | C16orf3 | NM_001214.3 | V44A | missense | rs61740023 | 0.009642 | - | 8.741 | - |  |  |  |  |  |  |  |  |
| chr5 | >40853031" | CARD6 | NM_032587 | R533C | missense |  | 0.0003068 | 0.0016 | 25.9 | 0.297 |  |  |  |  |  |  |  |  |
| chr21 | >44483184" | CBS | NM_001178008 | I278T | missense | rs5742905 | - | 0.0002 | 22.2 | 0.740 |  |  |  |  |  |  |  |  |
| chr1 | >17263214" | CROCC | NM_014675 | R347W | missense |  | 0.01899 | 0.0112 | 23.6 | 0.087 |  |  |  |  |  |  |  |  |
| chr4 | >191003044" | DUX4L4 | NM_001177376.2 | R258W | missense | rs370569001 | - | - | 16.35 | - |  |  |  |  |  |  |  |  |
| chrX | >55172659" | FAM104B | NM_138362.3 | I69T | missense | rs5018687 | - | - | 6.440 | 0.021 |  |  |  |  |  |  |  |  |
| chr1 | >152278856" | FLG | NM_002016.1 | S2836R | missense | rs11582087 | - | - | - | - |  |  |  |  |  |  |  |  |
| chr11 | >49208267" | FOLH1 | NM_001014986 | R190W | missense | rs75111588 | - | - | - | - |  |  |  |  |  |  |  |  |
| chr5 | >156479571" | HAVCR1 | NM_012206 | M158I | missense | rs75340804 | - | - | 0.041 | 0.128 |  |  |  |  |  |  |  |  |
| chr15 | >28447562" | HERC2 | NM_004667 | S2471P | missense |  | 0.04891 | - | 16.33 | 0.110 |  |  |  |  |  |  |  |  |
| chr6 | >32714125" | HLA-DQA2 | NM_020056 | Q241R | missense |  | 1.652e-05 | - | 0.009 | 0.066 |  |  |  |  |  |  |  |  |
| chr6 | >32549588" | HLA-DRB1 | NM_002124 | S133L | missense | rs117994013 | - | - | - | - |  |  |  |  |  |  |  |  |
| chr16 | >70954691" | HYDIN | NM_001270974 | K2530E | missense | rs1798528 | - | - | - | - |  |  |  |  |  |  |  |  |
| chr16 | >70891640" | HYDIN | NM_001270974.1 | K4088R | missense | rs1774416 | - | - | - | - |  |  |  |  |  |  |  |  |
| chr16 | >70908771" | HYDIN | NM_001270974.1 | P3537A | missense |  | - | - | - | - |  |  |  |  |  |  |  |  |
| chr16 | >70926334" | HYDIN | NM_001270974.1 | T3116R | missense | rs1774423 | - | - | - | - |  |  |  |  |  |  |  |  |
| chr1 | >117146504" | IGSF3 | NM_001007237 | R456C | missense | rs61786577 | - | - | - | - |  |  |  |  |  |  |  |  |
| chr20 | >47991056" | KCNB1 | NM_004975.2 | S347R | missense |  | - | - | - | - |  |  |  |  |  |  |  |  |
| chr19 | >55358686" | KIR2DS4 | NM_012314.3 | K247N | Missense | rs112697729 | - | - | - | - |  |  |  |  |  |  |  |  |
| chr17 | >39471763" | KRTAP17-1 | NM_031964.1 | G47D | missense | rs74252500 | - | - | 8.622 | - |  |  |  |  |  |  |  |  |
| chr17 | >39274364" | KRTAP4-11 | NM_033059 | R68S | missense | rs425784 | 0.0006367 | - | 21.3 | 0.116 |  |  |  |  |  |  |  |  |
| chr17 | >39346622" | KRTAP9-1 | NM_001190460 | C162S | missense | rs79470847 | - | - | - | - |  |  |  |  |  |  |  |  |
| chr19 | >55105722" | LILRA1 | NM_001278318.1 | V5L | missense |  | - | - | - | - |  |  |  |  |  |  |  |  |
| chr7 | >100677279" | MUC17 | NM_001040105 | S861T | missense | rs76184171 | - | - | - | - |  |  |  |  |  |  |  |  |
| chr7 | >100677285" | MUC17 | NM_001040105 | G863E | missense | rs74852422 | - | - | - | - |  |  |  |  |  |  |  |  |
| chr7 | >100678481" | MUC17 | NM_001040105 | T1262S | missense | rs73168389 | - | - | - | - |  |  |  |  |  |  |  |  |
| chr7 | >100678560" | MUC17 | NM_001040105 | T1288K | missense | rs77199586 | 0.07878 | - | 0.001 | 0.124 |  |  |  |  |  |  |  |  |
| chr7 | >100678568" | MUC17 | NM_001040105 | T1291S | missense | rs75492258 | - | - | - | - |  |  |  |  |  |  |  |  |
| chr7 | >100682624" | MUC17 | NM_001040105 | M2643L | missense |  | 0.06873 | - | 0.001 | 0.009 |  |  |  |  |  |  |  |  |
| chr6 | >30996959" | MUC22 | NM_001198815 | E1251K | missense | rs115182197 | 0 | - | 10.06 | 0.019 |  |  |  |  |  |  |  |  |
| chr7 | >100551035" | MUC3A | NM_005960 | S539T | missense | rs62483695 | - | - | - | - |  |  |  |  |  |  |  |  |
| chr7 | >100550795" | MUC3A | NM_005960 | T459I | missense |  | 0 | - | 1.183 | - |  |  |  |  |  |  |  |  |
| chr11 | >1267825" | MUC5B | NM_002458.2 | A3239P | missense | rs200304875 | - | - | - | - |  |  |  |  |  |  |  |  |
| chr11 | >1267852" | MUC5B | NM_002458.2 | A3248T | missense | rs201342042 | - | - | - | - |  |  |  |  |  |  |  |  |
| chr11 | >1017498" | MUC6 | NM_005961 | S1768T | missense | rs67507057 | - | - | - | - |  |  |  |  |  |  |  |  |
| chr11 | >1017746" | MUC6 | NM_005961 | L1685F | missense | rs78848170 | - | - | - | - |  |  |  |  |  |  |  |  |
| chr11 | >1018419" | MUC6 | NM_005961 | T1461I | missense |  | 0.2516 | - | 0.087 | 0.013 |  |  |  |  |  |  |  |  |
| chr11 | >1017084" | MUC6 | NM_005961 | P1906L | missense | rs34649796 | 0.2516 | - | 0.087 | 0.013 |  |  |  |  |  |  |  |  |
| chr11 | >1017498" | MUC6 | NM_005961 | S1768T | missense | rs67507057 | - | - | - | - |  |  |  |  |  |  |  |  |
| chr1 | >145281656" | NOTCH2NL | NM_203458 | T196S | missense | rs4649852 | - | - | - | - |  |  |  |  |  |  |  |  |
| chr10 | >47087078" | NPY4R | NM_005972.5 | A99S | missense |  | 0.2011 | - | 24.1 | 0.344 |  |  |  |  |  |  |  |  |
| chr11 | >48387237" | OR4C5 | ENST00000319813 | V261I | missense | rs72898877 | 0.2699 | - | 21.9 | 0.063 |  |  |  |  |  |  |  |  |
| chr8 | >101721839" | PABPC1 | NM_002568 | V365L | missense |  | 0.0153 | - | 0.025 | 0.009 |  |  |  |  |  |  |  |  |
| chr7 | >72413581" | POM121 | NM_172020 | V752I | missense | rs71554687 | - | - | - | - |  |  |  |  |  |  |  |  |
| chr7 | >72413593" | POM121 | NM_001257190 | Y756H | missense | rs71554688 | - | - | - | - |  |  |  |  |  |  |  |  |
| chr7 | >72413581" | POM121 | NM_172020 | V752I | missense | rs71554687 | - | - | - | - |  |  |  |  |  |  |  |  |
| chr7 | >72413593" | POM121 | NM_001257190 | Y756H | missense | rs71554688 | - | - | - | - |  |  |  |  |  |  |  |  |
| chr1 | >89449483" | RBMXL1 | NM_019610 | K9N | missense | rs74100106 | - | - | - | - |  |  |  |  |  |  |  |  |
| chr19 | >56283297" | RFPL4AL1 | NM_001277397 | D43N | missense | rs75204738 | 0.05604 | - | 21.4 | 0.467 |  |  |  |  |  |  |  |  |
| chr14 | >60074085" | RTN1 | NM_206852 | A63T | missense |  | - | - | - | - |  |  |  |  |  |  |  |  |
| chr10 | >46967616" | SYT15 | NM_031912.4 | S154L | missense | rs74128855 | 3.98e-05 | 0.0002 | 24.5 | 0.200 |  |  |  |  |  |  |  |  |
| chr11 | >1856582" | SYT8 | NM_138567.3 | C65G | missense |  | - | - | - | - |  |  |  |  |  |  |  |  |
| chr12 | >11183722" | TAS2R31 | NM_176885 | F71L | missense | rs78562467 | - | - | - | - |  |  |  |  |  |  |  |  |
| chr16 | >24788455" | TNRC6A | NM_014494 | P122Q | missense |  | 0.3414 | - | 0.004 | 0.118 |  |  |  |  |  |  |  |  |
| chr1 | >117660667" | TRIM45 | NM_025188 | L404P | missense |  | - | - | - | - |  |  |  |  |  |  |  |  |
| chr19 | >44891043" | ZNF285 | NM_001291491 | P300Q | missense | rs77661661 | 3.992e-06 | - | 24.0 | 0.293 |  |  |  |  |  |  |  |  |
| chr19 | >9271435" | ZNF317 | NM_020933.4 | R372C | missense |  | 0.03992 | - | 2.076 | - |  |  |  |  |  |  |  |  |
| chr3 | >75786211" | ZNF717 | NM_001290209 | S805P | missense | rs77715040 | 0.0003696 | - | 22.8 | 0.201 |  |  |  |  |  |  |  |  |
| chr3 | >75786759" | ZNF717 | NM_001290208 | K672R | missense | rs75759940 | - | - | 24.4 | 0.080 |  |  |  |  |  |  |  |  |
| chr3 | >75788199" | ZNF717 | NM_001128223 | H192R | missense | rs77110669 | 2.135e-05 | - | 14.79 | 0.160 |  |  |  |  |  |  |  |  |
| chr3 | >75787620" | ZNF717 | NM_001290208 | H385R | missense | rs75737034 | 0.0421 | - | 22.4 | 0.124 |  |  |  |  |  |  |  |  |
| chr6 | >32714164" | ZNF717 | NM_020056 | L254P | missense | rs115121776 | - | - | 3.463 | 0.066 |  |  |  |  |  |  |  |  |
| chr7 | >72081774" | ZNF717 | ENST00000435769 | C557R | missense | rs3015854 | - | - | - | - |  |  |  |  |  |  |  |  |
| chr3 | >75786942" | ZNF717 | NM_001290209 | R561I | missense | rs2918517 | - | - | - | - |  |  |  |  |  |  |  |  |
| chr3 | >75788158" | ZNF717 | NM_001290209 | L156V | missense | rs3009004 | - | - | 22.1 | 0.311 |  |  |  |  |  |  |  |  |
| chr3 | >75786687" | ZNF717 | NM_001290208 | L696H | missense | rs80214016 | 0.3987 | - | 22.2 | 0.438 |  |  |  |  |  |  |  |  |
| chr3 | >75787199" | ZNF717 | NM_001290209 | H475Q | missense |  | - | - | 15.27 | 0.155 |  |  |  |  |  |  |  |  |
| chr3 | >75788068" | ZNF717 | NM_001128223 | F236V | missense | rs74357986 | - | - | - | - |  |  |  |  |  |  |  |  |
| chr3 | >75786252" | ZNF717 | NM_001290208 | P841H | missense | rs79138891 | - | - | 0.876 | 0.083 |  |  |  |  |  |  |  |  |
| chr3 | >75787405" | ZNF717 | NM_001290209 | G407R | missense | rs73843014 | - | - | - | - |  |  |  |  |  |  |  |  |
| chr3 | >75787081" | ZNF717 | NM_001128223.1 | C565S | missense | rs77378861 | - | - | - | - |  |  |  |  |  |  |  |  |

**Supplementary Table 1**. All de novo missense variants.
