# Supplementary material for: A Pilot Study on Early-Onset Schizophrenia Reveals the Implication of Wnt, Cadherin and Cholecystokinin Receptor Signaling in Its Pathophysiology
Source: Front Genet. 2021 Dec 17;12:792218. doi: 10.3389/fgene.2021.792218 (PMC8719199; doi:10.3389/fgene.2021.792218)
Supplement: Supplementary file 4 [file Table2.DOCX]

| Chr | position | Gene | Ref seq | Protein variant | Variant type | Rs |
| --- | --- | --- | --- | --- | --- | --- |
| Fam 1 |  |  |  |  |  |  |
| chr3 | >75786942" | ZNF717 | NM_001290209 | R561I | missense | rs2918517 |
| chr3 | >75788158" | ZNF717 | NM_001290209 | L156V | missense | rs3009004 |
| Fam2 |  |  |  |  |  |  |
| chr3 | >75786175" | ZNF717 | NM_001290208 | E867* | nonsense | rs78135954 |
| chr3 | >75786687" | ZNF717 | NM_001290208 | L696H | missense | rs80214016 |
| chr3 | >75787199" | ZNF717 | NM_001290209 | H475Q | missense |  |
| Fam3 |  |  |  |  |  |  |
| chr3 | >75788068" | ZNF717 | NM_001128223 | F236V | missense | rs74357986 |
| chr3 | >75788199" | ZNF717 | NM_001128223 | H192R | missense | rs77110669 |
| Fam4 |  |  |  |  |  |  |
| chr3 | >75786252" | ZNF717 | NM_001290208 | P841H | missense | rs79138891 |
| chr3 | >75787405" | ZNF717 | NM_001290209 | G407R | missense | rs73843014 |
| chr3 | >75787620" | ZNF717 | NM_001290208 | H385R | missense | rs75737034 |
| Fam5 |  |  |  |  |  |  |
| chr3 | >75786041" | ZNF717 | NM_001128223.1 | S911X | frameshift | rs77747132&rs150497643 |
| Fam7 |  |  |  |  |  |  |
| chr3 | >75786035" | ZNF717 | NM_001128223.1 | F915FX | frameshift | rs149076283 |
| chr3 | >75787081" | ZNF717 | NM_001128223.1 | C565S | missense | rs77378861 |
| chr3 | >75787266" | ZNF717 | NM_001128223.1 | TG504TX | frameshift | rs776210532 |
| chr3 | >75788403" | ZNF717 | NM_001128223.1 | T124NX | frameshift |  |
| Fam1 |  |  |  |  |  |  |
| chr11 | >1017444" | MUC6 | NM_005961 | | Exon |  |
| chr11 | >1017498" | MUC6 | NM_005961 | S1768T | missense |  |
| chr11 | >1017746" | MUC6 | NM_005961 | L1685F | missense | rs78848170 |
| Fam2 |  |  |  |  |  |  |
| chr11 | >1016989" | MUC6 | NM_005961 | | Exon |  |
| chr11 | >1018419" | MUC6 | NM_005961 | T1461I | missense |  |
| Fam3 |  |  |  |  |  |  |
| chr11 | >1017084" | MUC6 | NM_005961 | P1906L | missense | rs34649796 |
| Fam4 |  |  |  |  |  |  |
| chr11 | >1017498" | MUC6 | NM_005961 | S1768T | missense |  |
| Fam5 |  |  |  |  |  |  |
| chr11 | >1017443" | MUC6 | NM_005961.2 | TT1786T | Inframe deletion |  |
| RBMXL3 |  |  |  |  |  |  |
| Fam1 |  |  |  |  |  |  |
| chrX | >114425105" | RBMXL3 | NM_001145346 | | exon  deletion |  |
| Fam3 |  |  |  |  |  |  |
| chrX | >114425105" | RBMXL3 | NM_001145346 | | exon  deletion |  |
| Fam4 |  |  |  |  |  |  |
| chrX | >114425105" | RBMXL3 | NM_001145346 | | exon  deletion |  |
| ACAN |  |  |  |  |  |  |
| Fam2 |  |  |  |  |  |  |
| chr15 | >89399701" | ACAN | NM_001135 | D1295E | missense |  |
| Fam3 |  |  |  |  |  |  |
| chr15 | >89399758" | ACAN | NM_013227 | D1314E | missense |  |
| Fam4 |  |  |  |  |  |  |
| chr15 | >89400023" | ACAN | NM_013227 | T1403A | missense | rs12899191 |
| PABPC1 |  |  |  |  |  |  |
| Fam1 |  |  |  |  |  |  |
| chr8 | >101721839" | PABPC1 | NM_002568 | V365L | missense |  |
| Fam2 |  |  |  |  |  |  |
| chr8 | >101721933" | PABPC1 | NM_002568 | | exon deletion |  |
| Fam3 |  |  |  |  |  |  |
| chr8 | >101719035" | PABPC1 | NM_002568 | | intron+splice intron |  |
|  |  |  |  |  |  |  |
| FRG1 |  |  |  |  |  |  |
| Fam1 |  |  |  |  |  |  |
| chr4 | >190878556" | FRG1 | NM_004477 | | Exon deletion |  |
| Fm2 |  |  |  |  |  |  |
| chr4 | >190864422" | FRG1 | NM_004477 | | exon insertion |  |
| Fam3 |  |  |  |  |  |  |
| chr4 | >190862999" | FRG1 | NM_004477 | | intron deletion |  |

**Supplementary Table 2.** De novo variants in genes common for at least three families.
