# Supplementary material for: A Pilot Study on Early-Onset Schizophrenia Reveals the Implication of Wnt, Cadherin and Cholecystokinin Receptor Signaling in Its Pathophysiology
Source: Front Genet. 2021 Dec 17;12:792218. doi: 10.3389/fgene.2021.792218 (PMC8719199; doi:10.3389/fgene.2021.792218)
Supplement: Supplementary file 5 [file Table3.DOCX]

| **STRING analysis statistics** | |
| --- | --- |
| Number of nodes: 463 | Avg. local clustering coefficient: 0.352 |
| Number of edges: 473 | Expected number of edges: 384 |
| Average node degree: 2.04 | PPI enrichment P-value: 7.08e-06 |

**Supplementary Table 3.** Representation of statistical results of STRING analysis.
